# Cancer Genetics Education in a Low- to Middle-Income Country: Evaluation of an Interactive Workshop for Clinicians in Kenya

**DOI:** 10.1371/journal.pone.0129852

**Published:** 2015-06-02

**Authors:** Jessica A. Hill, Su Yeon Lee, Lucy Njambi, Timothy W. Corson, Helen Dimaras

**Affiliations:** 1 Department of Ophthalmology and Vision Sciences, The Hospital for Sick Children, Toronto, Canada; 2 Laboratory Medicine and Pathobiology Program, University of Toronto, Toronto, Canada; 3 Department of Ophthalmology & Vision Sciences, University of Toronto, Toronto, Canada; 4 Department of Ophthalmology, University of Nairobi, Nairobi, Kenya; 5 Department of Human Pathology, University of Nairobi, Nairobi, Kenya; 6 Department of Ophthalmology, Indiana University School of Medicine, Indianapolis, Indiana, United States of America; 7 Department of Biochemistry and Molecular Biology, Indiana University School of Medicine, Indianapolis, Indiana, United States of America; 8 Department of Pharmacology and Toxicology, Indiana University School of Medicine, Indianapolis, Indiana, United States of America; Cancer Research Centre of Lyon, FRANCE

## Abstract

**Background:**

Clinical genetic testing is becoming an integral part of medical care for inherited disorders. While genetic testing and counseling are readily available in high-income countries, in low- and middle-income countries like Kenya genetic testing is limited and genetic counseling is virtually non-existent. Genetic testing is likely to become widespread in Kenya within the next decade, yet there has not been a concomitant increase in genetic counseling resources. To address this gap, we designed an interactive workshop for clinicians in Kenya focused on the genetics of the childhood eye cancer retinoblastoma. The objectives were to increase retinoblastoma genetics knowledge, build genetic counseling skills and increase confidence in those skills.

**Methods:**

The workshop was conducted at the 2013 Kenyan National Retinoblastoma Strategy meeting. It included a retinoblastoma genetics presentation, small group discussion of case studies and genetic counseling role-play. Knowledge was assessed by standardized test, and genetic counseling skills and confidence by questionnaire.

**Results:**

Knowledge increased significantly post-workshop, driven by increased knowledge of retinoblastoma causative genetics. One-year post-workshop, participant knowledge had returned to baseline, indicating that knowledge retention requires more frequent reinforcement. Participants reported feeling more confident discussing genetics with patients, and had integrated more genetic counseling into patient interactions.

**Conclusion:**

A comprehensive retinoblastoma genetics workshop can increase the knowledge and skills necessary for effective retinoblastoma genetic counseling.

## Introduction

Diagnostic genetic testing is quickly becoming integrated into the standard of care for many inherited disorders. The complexity of the genetic information received coupled with the heightened emotion of receiving a genetic diagnosis is best served by genetic counseling services, which help patients cope with new information and make informed decisions given their personal circumstances [1]. Recent progress in the development of genomic methods, such as next generation sequencing, has decreased cost and thus increased accessibility of genetic and genomic testing, making the need for adequate genetic counseling all the more pressing [2]. However, the specialized training required to interpret complex genetic data is a significant barrier to the implementation of counseling services [3,4].

Access to genetic testing and specialized genetic counseling varies globally. These services are largely available in high-income countries, and typically provided by an expert in the field, such as a medical geneticist or a genetic counselor. In low- and middle-income countries (LMICs) such as Kenya, clinical genetic testing for a disease like the early childhood eye cancer retinoblastoma is unavailable locally and the burden of genetic counseling falls to physicians who have not received specialized training in medical genetics [5]. With advances in technology, genetic testing is likely to become widespread in Kenya within the next decade, yet there has not been a concomitant increase in genetic counseling resources. In order to effectively and ethically implement genetic testing, it must be coupled with counseling that is in accordance with local religious, social and cultural views [6]. This will require training health care workers in the interpretation of genetic testing and in the implementation of socioculturally sensitive counseling services.

Retinoblastoma is an aggressive early childhood eye cancer and the first cancer for which the genetic basis was determined [7,8]. Retinoblastoma is initiated by loss of function of the tumor suppressor gene *RB1* [9]. With early detection and proper care, life can be preserved, but a diagnosis of retinoblastoma comes with long-term medical implications for both the affected individual and members of his/her family. Nearly half of these affected individuals are at increased risk of second cancers, and family members like siblings and future offspring may be susceptible [10]. Genetic testing can resolve the likelihood of these possibilities.

In Kenya, the incidence of retinoblastoma (1 in 17,030 live births) is comparable to that observed worldwide [11]. The relatively high Kenyan birth rate [12] results in a large burden of disease with an estimated 90 expected new retinoblastoma cases per year, in contrast to the 29 new cases expected annually in a country with a comparable population size, Spain. Although Kenya faces several challenges in implementing effective care for retinoblastoma patients [10,13], a proactive approach to deliver evidence-based care for retinoblastoma patients and families has been taken with the formation of the Kenyan National Retinoblastoma Strategy group (KNRbS), a multidisciplinary team of clinicians, support workers and retinoblastoma-affected family members dedicated to improving retinoblastoma-related outcomes in East Africa [14], and with the publication of guidelines for retinoblastoma care endorsed by the Kenyan Ministry of Health [15]. Retinoblastoma genetic counseling can be delivered in the presence or absence of genetic testing, with a more conservative approach for surveillance of families and affected individuals in the absence of genetic testing. In the Kenyan context, this means that clinicians delivering retinoblastoma counseling need to be educated for both scenarios. Integrating retinoblastoma genetic testing and counseling knowledge is necessary for delivery of best care in an era of burgeoning genetic testing.

Our aim was thus to determine if a comprehensive and interactive workshop on retinoblastoma genetics could build capacity in retinoblastoma genetic counseling in the healthcare workforce in Kenya. Here we present our experience in designing, implementing and testing an intervention aimed at increasing capacity in genetic knowledge and confidence in genetic counseling, for physicians and other medical professionals caring for retinoblastoma patients and their families.

## Methods

### Research Ethics

**Fig 1:**
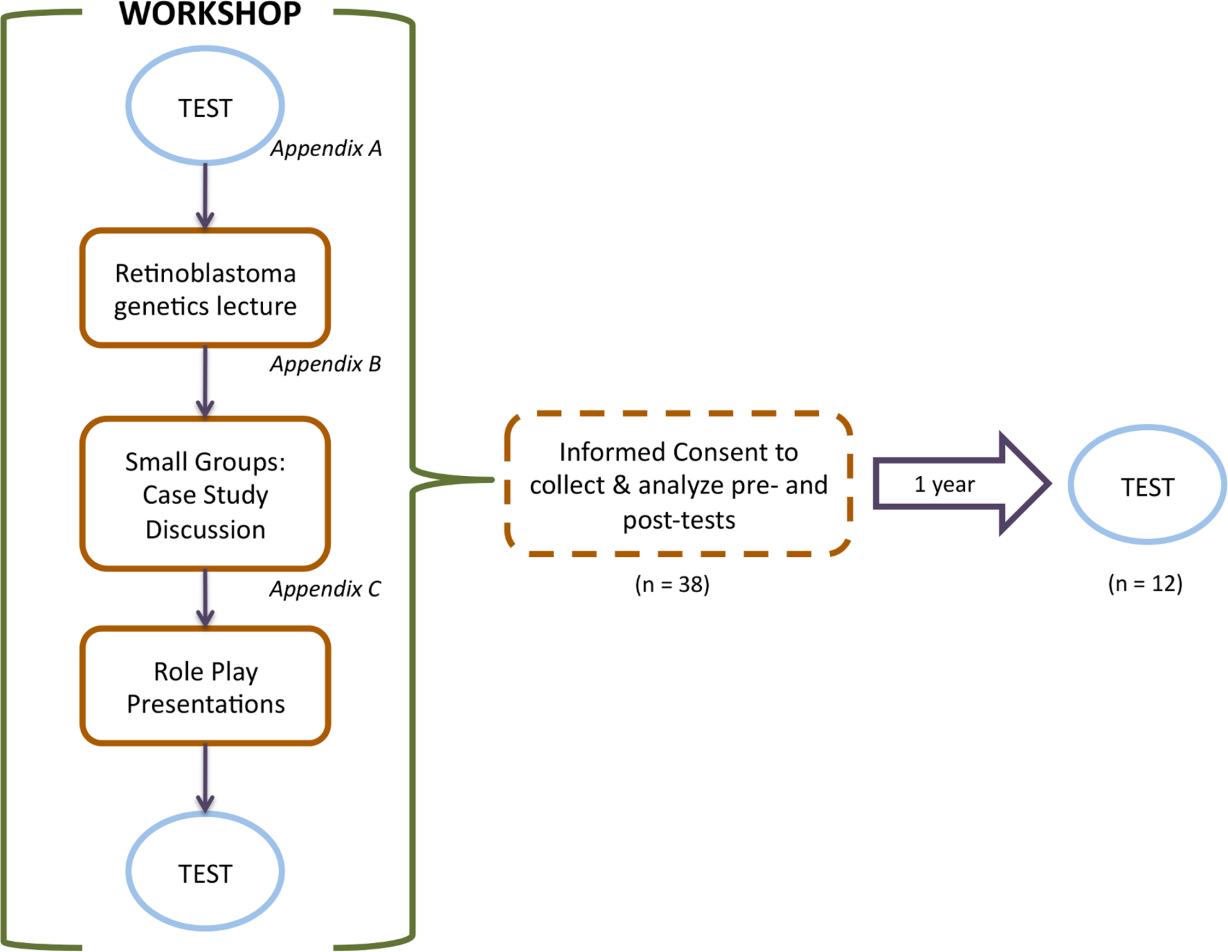
Sequence of events of training workshop and testing. The workshop consisted of a knowledge test before and after a lecture on retinoblastoma genetics, small group discussions of case studies, and role-play presentations of case study scenarios. Informed consent was obtained from workshop participants who agreed to have their tests included in this analysis, and tested 1-year after the workshop.

Ethics approval was obtained from the University of Toronto Research Ethics Board. Workshop participants were asked to sign a consent form to contribute their anonymous test scores to this analysis. All research was conducted according to the principles expressed in the Declaration of Helsinki. The researcher (HD) gave a brief outline of the purpose and methodology of the study, the potential benefits and/or harms of the study, and explained that participation was completely voluntary (Fig 1).

### Participants

The retinoblastoma genetics workshop took place in September 2013 in Eldoret, Kenya, during an afternoon session of the annual KNRbS meeting. The workshop participants were KNRbS meeting attendees: ophthalmologists, pathologists, oncologists, ophthalmic clinical officers and nurses. The workshop was attended by 55 individuals, and informed consent was obtained from 38 participants for use of their tests and permission to re-test after a year. The subsequent one-year post-workshop knowledge retention test was conducted at the September 2014 KNRbS meeting held in Nairobi, Kenya. The one-year post-workshop test was administered to consented attendees who had participated in the workshop the previous year (n = 12).

### Workshop composition

The composition of the workshop is described in Fig 1. The workshop immediately followed the administration of the pre-test evaluation (S1 File). The retinoblastoma genetics workshop was composed of three sections: a retinoblastoma genetics lecture (S2 File), followed by a discussion of patient case studies and finally genetic counseling role-play of case studies (S3 File).

The retinoblastoma genetics lecture was written and presented by a retinoblastoma genetics specialist (author HD). The lecture was given using Microsoft PowerPoint and consisted of 38 slides (approximately 30 minutes of teaching). The lecture covered four general sections: 1) foundational retinoblastoma genetics, including heritability of mutations in *RB1* and their implications in family planning and risk for second cancers, 2) genetic testing for retinoblastoma, how it works and the spectrum of mutations that arise, 3) genetic counseling for retinoblastoma, with and without genetic testing, and 4) personal stories about genetics from retinoblastoma survivors in attendance, followed by case studies.

Participants were subsequently divided into five groups. Care was taken to ensure groups were composed of multidisciplinary members. Each group was assigned a case study that depicted a clinical scenario and was accompanied by a pedigree (diagram indicating the familial history of retinoblastoma). The case studies encompassed family planning and retinoblastoma diagnostics in the absence of genetic testing. Each case study was a variation on the following format: an individual or family visiting a doctor, inquiring about likelihood of retinoblastoma affecting newborns/future children given a certain family history with retinoblastoma. The cases reflected real-life scenarios where clinicians would have varying degrees of certainty regarding the inheritance of retinoblastoma in future offspring.

Participants were asked to discuss a series of follow-up questions related to their case study. The discussion questions were as follows: 1) How would you counsel these family members? 2) What are the key concepts regarding genetics and inheritance present in this case? 3) If genetic testing were available, what additional information could you learn? Would this change the message for the patient/patient’s family? Would this affect patient care? 4) What are some of the key issues you think should be taken into consideration when counseling patient families and retinoblastoma survivors in Kenya? A retinoblastoma genetics expert (a physician or researcher with ample experience in retinoblastoma genetics) was randomly assigned to each group to facilitate discussion. The possibility of contacting these retinoblastoma genetics experts after the workshop was available to participants.

Following the small group discussion period (approximately 30 minutes), group members were asked to assign themselves roles of individuals from the case study (eg. treating doctor, parent, survivor, nurse, genetic counselor) and to act out how they would manage the genetic counseling session. Each group was allotted 5–10 minutes to present their role-play scenario to the broader group. This was followed by a brief discussion amongst all participants on what aspects of the skit were most valuable and what could be improved upon in the future.

### Retinoblastoma genetics curriculum evaluation

**Fig 2:**
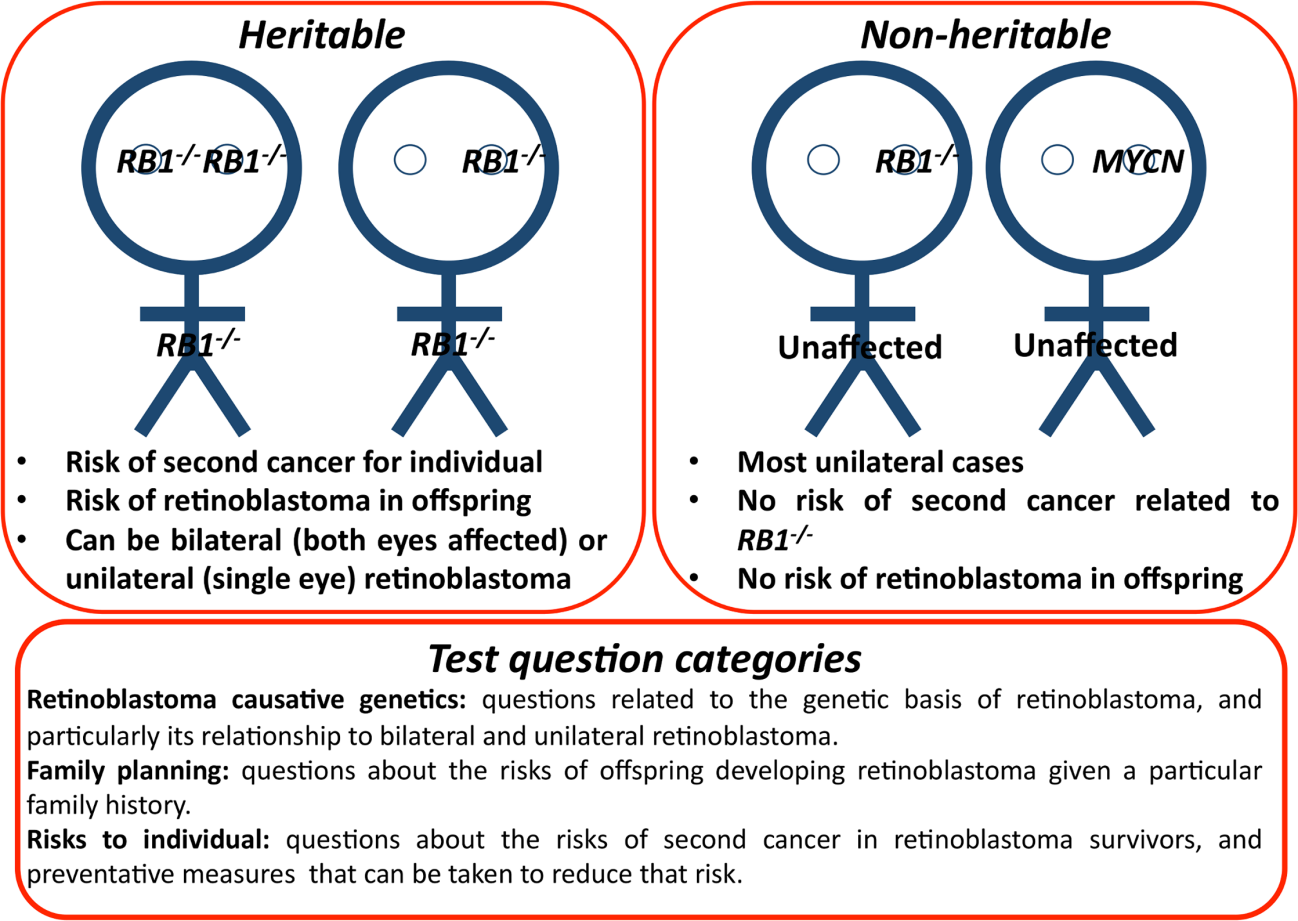
An introduction to retinoblastoma genetics. Retinoblastoma is initiated by loss-of-function of the tumor suppressor gene *RB1* (*RB1* ^*-/-*^) in one eye (unilateral) or both eyes (bilateral). There are different implications for care for different scenarios. Notably, a rare cause of non-heritable retinoblastoma is unrelated to *RB1*, amplification of the gene *MYCN*.

The impact of the workshop on retinoblastoma genetics knowledge was determined by administering a standardized test immediately post- and one-year post-workshop. The same test was used to establish the baseline level of knowledge prior to the workshop. The test consisted of ten multiple-choice or true/false questions. Some questions had multiple correct answers such that the maximum score achievable was 20. Test questions covered three broad areas: retinoblastoma causative genetics (Q1—Q5), family planning (Q6—Q7) and risks to individual (Q8—Q10); see Fig 2. Some test questions referenced technologies new to retinoblastoma care that are currently unavailable in Kenya. Differences in test scores between testing sessions were evaluated by ANOVA, and subsequent Tukey post-hoc test.

As part of the test immediately post-workshop, feedback on the value of the workshop was solicited from participants. One-year post-workshop, participants were asked whether the workshop impacted their daily performance treating and counseling retinoblastoma patients, and whether the role-play section of the workshop was useful. Participants were also asked for any additional feedback.

## Results

### Baseline level of retinoblastoma genetics knowledge

We first examined the pre-workshop test responses by category to determine the baseline level of knowledge of retinoblastoma genetics and implications for the patient and his/her family (Fig 1). The mean test score was 72% (14.4/20). Examining questions by category revealed that there was no difference in score between categories (ANOVA, F_2,17_ = 0.5, P = 0.61), where the mean score was 78.9% for retinoblastoma causative genetics, 71.5% for family planning and 68.5% for risks to individual. There was, however, substantial variation in successful responses between questions. The greatest range in responses was 100% correct (Question 1, a basic retinoblastoma causative genetics question) to 21% correct (Question 8a, a more sophisticated question about second cancer risk).

### Multidisciplinary groups role play retinoblastoma case studies

Workshop participants were divided into five multidisciplinary groups and assigned a clinical case study to discuss and enact. Although the case studies varied with respect to the amount of information the participants could extract, common points emerged between presentations. First, the participant’s career experience informed his/her counseling during the role-play. For example, the doctors in the role-play asked detailed questions about family history with respect to retinoblastoma and other eye loss that could be attributed to retinoblastoma. In contrast, a child life expert integrated child-oriented care into her counseling. She played with the affected ‘baby’ and gave him toys to distract him, to help the ‘parents’ better focus on the counseling message. In contrast, her role-play counseling counterpart played by an ophthalmologist, focused on information for parents which was more traditionally medical and based in retinoblastoma causative genetics, etc. Clinicians playing the part of adult family members of retinoblastoma survivors (or adult survivors themselves) raised issues that they perceived as commonly raised by parents of retinoblastoma survivors, such as attempting to assign blame for heritable retinoblastoma, asking if a traditional healer should be consulted and asking about the time and financial constraints of treatment. These prompted discussion from the rest of the audience after each presentation.

### Retinoblastoma genetics knowledge immediately post-workshop

**Fig 3:**
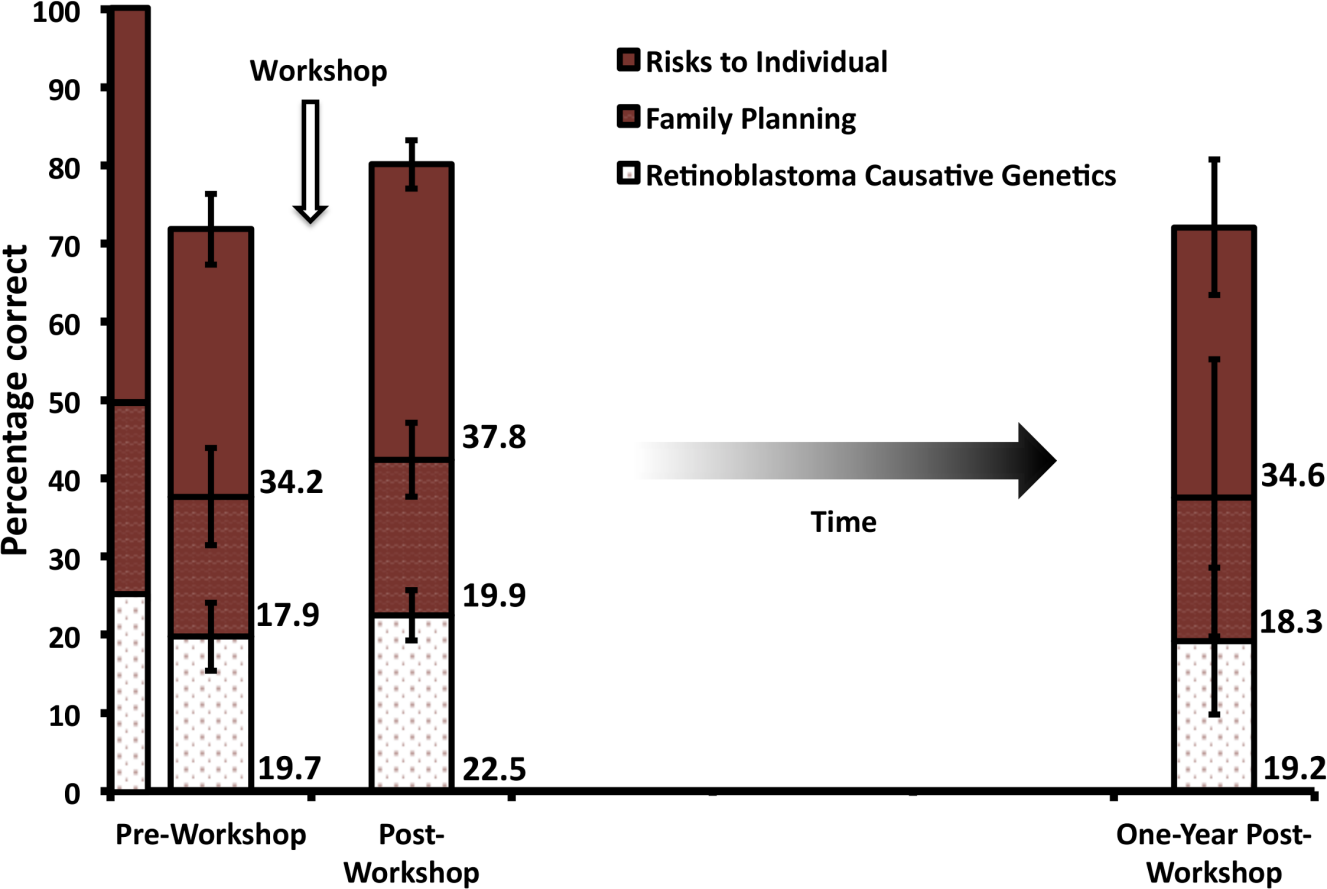
Mean test scores pre-, post- and one-year post-workshop. Scores are patterned according to the question category. The leftmost column displays what a perfect test would look like, where “risks to individual” questions encompass 50% of the test, and “family planning” and “retinoblastoma causative genetics” are the subject of 25% of the questions each. The weighted score for each category is listed on the right of the column for pre-, post- and one-year post-workshop scores. Error bars indicate standard deviation per category.

Overall, knowledge of retinoblastoma genetics significantly increased post-workshop (ANOVA, F_2,85_ = 4.6, P < 0.01, Tukey post-hoc test), from 72% (14.4/20) to 80% (16.0/20) (Fig 3). Determining the differences between scores by question category revealed a significant increase in score in retinoblastoma causative genetics questions (ANOVA, F_2,85_ = 1.04, P < 0.01, Tukey post-hoc test), but not in questions related to family planning (ANOVA, F_2,85_ = 1.04, P > 0.05), or risks to individual (ANOVA, F_2,85_ = 2.09, P > 0.05). Thus, the overall score increase was largely driven by the increase in knowledge in retinoblastoma causative genetics.

We sought to identify where the changes in response to individual questions were greatest between the pre-workshop and the post-workshop tests. Examining each question revealed that the greatest increase in score was found in questions related to recent discoveries in retinoblastoma genetics, such as Question 5 which concerns retinoblastoma caused by a rare and only recently appreciated alteration, overexpression of the oncogene *MYCN* [16]. The post-workshop responses had more correct answers for every question with the exception of Question 8b (regarding the risk of developing a second cancer in retinoblastoma survivors affected in one eye) and Question 10d (related whether risk of second cancers can be reduced by a new medical technology).

### Retinoblastoma genetics knowledge one-year post-workshop

Overall, the number of correct responses one-year post-workshop returned to 72% (14.4/20). However, likely due to increased variability, this result is not significantly different from either the pre- or post-workshop test scores (Tukey post-hoc test). Examining the change in score per type of question revealed that the mean score for the retinoblastoma diagnostic genetics questions was significantly lower than the post-workshop score, but no different from the pre-workshop score (Tukey post-hoc test). There was no significant difference in the one-year post-workshop score from either the pre- or post-workshop score for questions related to either family planning or risks to individual. Although the one-year post-workshop test score overall average was nearly identical to the pre-test score, there was variability in which questions participants answered correctly. In particular, participants had much greater success on Question 8a (a question about the risk of developing a second cancer in retinoblastoma survivors affected in both eyes) one-year post-workshop than they did pre-workshop.

### Participant feedback post-workshop

Participant comments indicated that they found the lecture material and the role-play useful and relevant to their practices. Respondents indicated that they found the material informative and appreciated the testimonials of affected families. One participant wrote *“The role play presented an opportunity to approach a complex subject in a manner that caused much reflection and review of broad and complex concepts and presented in a manner that made this better appreciated”*. Respondents also indicated that they would appreciate a handout summary of retinoblastoma genetics.

### Participant feedback one-year post-workshop

When asked one-year post-workshop whether the workshop had impacted their routine interactions with retinoblastoma patients and families, all participants responded positively. Several respondents elaborated, and most of those responses indicated that they now included genetic counseling in their practice. One respondent indicated that he/she had organized support groups for families affected. One respondent summarized the impact of the workshop as follows: *“Counselling helps parents with children with retinoblastoma to be aware of the possibility of having children with the same condition*. *And the children may also develop other tumours besides getting children with retinoblastoma*.*”* One-year post-workshop, all respondents indicated that the role-play part of the workshop was useful. Comments included that it *“helped in understanding better the genetics of retinoblastoma”* and *“helped us to understand the real life”*. Furthermore, 2014 KNRbS meeting attendees that had not attended the retinoblastoma genetics workshop the previous year (and were therefore excluded from these tests), indicated a desire for the retinoblastoma genetics workshop to be repeated for their benefit.

## Discussion

Here we employed a novel approach for capacity building in cancer genetics and genetic counseling amongst health professionals in Kenya. We conducted an afternoon workshop that consisted of a retinoblastoma genetics workshop, group discussion of retinoblastoma genetics case studies and case study role-play as genetic counseling practice. We found that the retinoblastoma genetics workshop increased participant knowledge of retinoblastoma genetics, largely due to an increase in knowledge in retinoblastoma causative genetics, but that the increase in knowledge was not retained one-year post-workshop. The workshop was favorably received by participants immediately post-workshop, and upon recollection one-year post-workshop.

Understanding retinoblastoma genetics is crucial for adequate care for retinoblastoma-affected families. Even in the absence of genetic testing, critical information about retinoblastoma heritability can be gleaned from family history (Fig 2). When this information is not appreciated and new family members are not vigilantly screened, poorer outcomes ensue. For example, a study focused exclusively on heritable retinoblastoma in developing countries demonstrated that despite a family history of the retinoblastoma, there was a delay in diagnosis in subsequent family members [17]. Furthermore, anecdotal evidence in Kenya suggests family history is not being acted upon, as second and third children with retinoblastoma are presenting with advanced-stage retinoblastoma despite the family’s prior experience with retinoblastoma. Even if risks are explained to families, some parents interpret the death or cancer experience of a first child to be caused by other factors such as witchcraft or hospital failure, and refuse to seek care for the next child. A better appreciation of the genetic information provided by family history is required for better outcomes, and approaches must vary with sociocultural context.

This study indicates that a genetics education intervention increased knowledge in retinoblastoma genetics (Fig 3). Although the one-year post-workshop test score was not significantly different from post-workshop score, this is likely due to the lack of power created by a small sample size of return participants at the 2014 KNRbS meeting one year after the retinoblastoma genetics workshop (n = 12). Indeed, the 2013 KNRbS meeting hosted several health care workers from neighboring countries such as Ethiopia, and local ophthalmology medical residents whose residency had been completed by 2014. These 2013 participants did not attend the 2014 KNRbS meeting.

Knowledge retention test scores may have been higher if the test was performed sooner than one-year post-workshop. However, one-year post-workshop is likely a better indicator of long-term retention than a shorter interval. This time frame also facilitated recruitment of participants by coupling the knowledge retention test with the 2014 KNRbS meeting, and gave participants more intervening opportunities to interact with retinoblastoma patients and their families than a shorter time frame would have, such that participants could form a more accurate impression of their own counseling abilities.

The increase in test score post-workshop was driven mainly by a significant increase in knowledge in retinoblastoma causative genetics. In particular, some participants may not have been familiar with a rare cause of retinoblastoma (amplification of *MYCN*) and the fact that some unilateral cases of retinoblastoma are heritable (see Fig 2). Awareness that unilateral retinoblastoma can be heritable is particularly important for clinicians when counseling affected families, because this diagnosis requires more vigilant surveillance of the affected individual, his/her future offspring and subsequent siblings. By the one-year post-workshop retention test, the score in retinoblastoma causative genetics knowledge returned to its baseline level, indicating that more frequent knowledge reinforcement is required.

The one-year post-workshop test score was identical to the pre-workshop test score of 72% correct. Participant responses on the one-year post-workshop test were lower than in the pre-workshop test in 11 marks of a possible 20. However, nearly twice as many respondents at the one-year post-workshop test answered Question 8a correctly. This question asks whether some retinoblastoma survivors affected in both eyes are at risk for second cancer (Fig 2). It is unclear why there was such an increase in correct responses to this question one-year post-workshop, yet not with other questions.

Our previous work evaluating the barriers to effective retinoblastoma care in Kenya revealed a need for genetic education of medical professionals [5]. Examination of the pre-test responses indicated areas of retinoblastoma genetics and counseling that require the most reinforcement are related to second cancer risk and prevention, and new advances in retinoblastoma diagnostics. Second cancers are a major cause of premature death amongst survivors of heritable retinoblastoma [18], but this may not have been a focus of Kenyan clinicians as the rate of survival from retinoblastoma has been low (26.6%) [19]. The lower test scores in questions related to new advances in retinoblastoma diagnostics and treatment are likely explained by the fact that these technologies are not currently accessible in Kenya. Intriguingly, when asked whether “choosing to not have more children” is an option for future offspring for a woman who has heritable retinoblastoma (Question 7d), only 57% responded positively. Although choosing to not have additional children would certainly limit the number of future offspring with retinoblastoma, a high proportion of respondents may have responded negatively because an ideal Kenyan family size includes several children [20].

Beyond an increase in retinoblastoma genetics knowledge, the case study role-play aimed to help health care workers develop genetic counseling skills to increase comfort and confidence in relaying complex genetic information to patients and their families. The multidisciplinary peer groups participating in role-play seemed to help health care workers to appreciate one another’s roles, such as the emphasis put on the affected child by a child life specialist when playing the role of a doctor during the case study role-play. Indeed, participants indicated that the unique case-study role-play aspect of our interactive workshop allowed them to appreciate the perspective of afflicted families. Furthermore, feedback from the one-year post-workshop survey indicated that some participants had incorporated more genetic counseling into the regular interactions with retinoblastoma patients and their families. Some participants indicated that they felt more confident in discussing retinoblastoma genetics with their patients and patient families. These were goals of the workshop that were not captured by a standardized test.

Participant response to the workshop was positive, and participant comments indicated that they welcomed the intervention. Despite the return of retinoblastoma genetics knowledge back to the baseline level in the intervening year, survey respondents indicated that they found the workshop informative, even at the one-year follow-up evaluation. These data suggest that the workshop should be repeated at more regular intervals to maintain retinoblastoma genetics knowledge, improve genetic counseling and communication skills, increase interprofessional interactions, increase confidence in counseling ability and retinoblastoma knowledge, and to provide exposure to new technologies in retinoblastoma care that may soon be available in Kenya. Furthermore, much knowledge was gained from the first implementation of this intervention. Future iterations of this workshop will aim to enhance the duration of retinoblastoma knowledge, for example by including take-home reference materials for participants such as summary reference leaflets that participants can refer to while counseling families. We will attempt to clarify concepts that participants scored poorly on, such as measures that can be taken to reduce the risk of second cancers in retinoblastoma survivors.

As genetic testing for cancer and other heritable diseases becomes more commonplace globally, there is an increasing appreciation of the need to train primary health care workers to be able to sensitively interpret and convey genetic information. Indeed, several interventions have been designed to train primary health care providers in clinical genetics in the US and Canada. As with our study, these researchers found their interventions increase knowledge of clinical cancer genetics and increased confidence in primary health care providers in their core genetics competency [21,22]. However, cancer care and education guidelines developed in resource-rich countries may have limited utility in low- and middle-income countries and must be carefully edited before considering implementation in these locations. Furthermore, complete patient care requires cultural aptitude as well as genetic aptitude [23]. For example, a cancer diagnosis in Kenya may be stigmatized [24]. Thus there is an increasing demand globally for genetic literacy and culturally sensitive counseling amongst primary health care workers as genetic testing is becoming more accessible. This workshop can serve as a template for an intervention to increase genetic literacy and counseling skills amongst clinicians, tailored to the appropriate sociocultural context.

Incorporating genetic testing and counseling in retinoblastoma care will help improve the outcomes for retinoblastoma patients and their families, and reduce the disparity in retinoblastoma survival between Kenya and developed countries. Adequately training health care professionals to interpret and sensitively convey this information to patient families is essential for its success at a component of patient care. Here we present a scalable strategy for increasing genetics knowledge and counseling skills amongst clinicians. Administering this strategy more broadly, and adapted for different genetic conditions and sociocultural contexts, would require access to increased funds and regulatory involvement, likely from local government or academia. Furthermore, as genetic testing becomes routine worldwide, the responsibility of test administration and interpretation may fall less to specialized genetic counselors and more to primary health care providers. Thus, new methods of training the primary health care workforce in providing genetic services in LMICs may ultimately inform practice in high-income countries.

## Conclusions

The retinoblastoma genetics interactive workshop was positively received by participants. Immediately post-workshop, retinoblastoma genetics knowledge was improved however knowledge was not retained one-year post-workshop suggesting the workshop should be conducted more frequently. Encouragingly, one-year post-workshop, participants self-rated their retinoblastoma genetic counseling skills as improved, indicating that genetic counseling may be successfully integrated into health care professionals’ current duties.

## Supporting Information

S1 File Retinoblastoma genetics workshop test. (PDF)

S2 File Retinoblastoma genetics lecture. (PDF)

S3 File Retinoblastoma case studies. (PDF)
