# Supplementary material for: Cancer Genetics Education in a Low- to Middle-Income Country: Evaluation of an Interactive Workshop for Clinicians in Kenya
Source: PLoS One. 2015 Jun 2;10(6):e0129852. doi: 10.1371/journal.pone.0129852 (PMC4452713; doi:10.1371/journal.pone.0129852)
Supplement: S1 File — (PDF) [file pone.0129852.s001.pdf]

Pre-test #____

For all questions, check the most appropriate answer. If you do not know the answer, check 'I don't know'.

***

1. A bilateral retinoblastoma survivor has a risk of having a child with retinoblastoma.

☐ TRUE ☐ FALSE or: ☐ I don't know.

2. A unilateral retinoblastoma survivor may be at risk of having a child with retinoblastoma.

☐ TRUE ☐ FALSE or: ☐ I don't know.

3. Only bilateral retinoblastoma survivors with family history carry a germline *RB1* mutation.

☐ TRUE ☐ FALSE or: ☐ I don't know.

4. Some unilateral retinoblastoma survivors carry a germline *RB1* mutation.

☐ TRUE ☐ FALSE or: ☐ I don't know.

5. A survivor with *MYCN*-amplified retinoblastoma is at risk of having a child with retinoblastoma.

☐ TRUE ☐ FALSE or: ☐ I don't know.

6. Ruth had bilateral retinoblastoma when she was a child. Ruth has a newborn infant. Mutation testing revealed that the baby carries a germline *RB1* mutation. Will the baby develop retinoblastoma?

☐ No

☐ Yes, the baby will develop bilateral retinoblastoma.

☐ Yes, the baby will develop unilateral retinoblastoma

☐ Yes, the baby will develop either bilateral or unilateral retinoblastoma

☐ I don't know.

7. Ruth is concerned about her future pregnancies, and the chance that she may have more children at risk for retinoblastoma. What are some of her options for future offspring? Check all that apply, even if the options are not available in Kenya but available elsewhere.

☐ Prenatal *RB1* genetic diagnosis, early delivery and treatment of tumors as early/small as possible.

☐ Pre-implantation *RB1* genetic diagnosis (i.e. selection of an unaffected embryo for implantation).

☐ Clinic exams and EUAs by ophthalmologist as soon as possible after the baby's birth.

☐ Ruth could choose not to have any more children.

☐ I don't know.

8. Which retinoblastoma survivors are at increased risk of developing a second cancer? Check all that apply.

☐ Some bilateral survivors.

☐ All unilateral survivors.

☐ All survivors who had radiation treatment.

☐ All survivors with a germline *RB1* mutation.

☐ I don't know.

9. As an at-risk retinoblastoma survivor gets older, his/her risk of developing a second cancer decreases.

☐ TRUE ☐ FALSE ☐ I don't know.

10. How can retinoblastoma survivors reduce their risk of developing a second cancer? Check all that apply.

☐ Be vigilant about unexplained lumps, pain or skin changes.

☐ Eat a healthy diet rich in anti-oxidants.

☐ Avoid unnecessary radiation, such as X-ray and CT scan.

☐ Have annual full-body MRI.

☐ Avoid smoking and excess drinking.

☐ I don't know.

Post-test #____

For all questions, check the most appropriate answer. If you do not know the answer, check 'I don't know'.

***

1. A <u>bilateral</u> retinoblastoma survivor has a risk of having a child with retinoblastoma.

☐ TRUE ☐ FALSE or: ☐ I don't know.

2. A <u>unilateral</u> retinoblastoma survivor may be at risk of having a child with retinoblastoma.

☐ TRUE ☐ FALSE or: ☐ I don't know.

3. Only <u>bilateral</u> retinoblastoma survivors with family history carry a germline *RB1* mutation.

☐ TRUE ☐ FALSE or: ☐ I don't know.

4. Some <u>unilatera</u>l retinoblastoma survivors carry a germline *RB1* mutation.

☐ TRUE ☐ FALSE or: ☐ I don't know.

5. A survivor with *MYCN*-amplified retinoblastoma is at risk of having a child with retinoblastoma.

☐ TRUE ☐ FALSE or: ☐ I don't know.

6. Ruth had <u>bilateral</u> retinoblastoma when she was a child. Ruth has a newborn infant. Mutation testing revealed that the baby carries a germline *RB1* mutation. Will the baby develop retinoblastoma?

☐ No

☐ Yes, the baby will develop bilateral retinoblastoma.

☐ Yes, the baby will develop unilateral retinoblastoma

☐ Yes, the baby will develop either bilateral or unilateral retinoblastoma

☐ I don't know.

7. Ruth is concerned about her future pregnancies, and the chance that she may have more children at risk for retinoblastoma. What are some of her options for future offspring? Check <u>all</u> that apply, even if the options are not available in Kenya but available elsewhere.

☐ Prenatal *RB1* genetic diagnosis, early delivery and treatment of tumors as early/small as possible.

☐ Pre-implantation *RB1* genetic diagnosis (i.e. selection of an unaffected embryo for implantation).

☐ Clinic exams and EUAs by ophthalmologist as soon as possible after the baby's birth.

☐ Ruth could choose not to have any more children.

☐ I don't know.

8. Which retinoblastoma survivors are at increased risk of developing a second cancer? Check all that apply.

☐ Some bilateral survivors.

☐ All unilateral survivors.

☐ All survivors who had radiation treatment.

☐ All survivors with a germline *RB1* mutation.

☐ I don't know.

9. As an at-risk retinoblastoma survivor gets older, his/her risk of developing a second cancer decreases.

☐ TRUE ☐ FALSE ☐ I don't know.

10. How can retinoblastoma survivors reduce their risk of developing a second cancer? Check all that apply.

☐ Be vigilant about unexplained lumps, pain or skin changes.

☐ Eat a healthy diet rich in anti-oxidants.

☐ Avoid unnecessary radiation, such as X-ray and CT scan.

☐ Have annual full-body MRI.

☐ Avoid smoking and excess drinking.

☐ I don't know.

If you would like to receive your test scores and be re-tested in 2-3 months time, please provide your email address and we will send you an online link to the knowledge retention test:

Email: ____
