# Supplementary material for: Cancer Genetics Education in a Low- to Middle-Income Country: Evaluation of an Interactive Workshop for Clinicians in Kenya
Source: PLoS One. 2015 Jun 2;10(6):e0129852. doi: 10.1371/journal.pone.0129852 (PMC4452713; doi:10.1371/journal.pone.0129852)
Supplement: S2 File — (PDF) [file pone.0129852.s002.pdf]

Retinoblastoma Genetics Workshop

Helen Dimaras, PhD

13 September 2013

6th Annual KNRbS Meeting

Pop quiz!

10 questions, 5 minutes.

Outline

1. Retinoblastoma is a Genetic Disease
2. Retinoblastoma Genetic Testing
3. Genetic Counseling for Retinoblastoma
4. Personal Stories: RB Genetics

1. Retinoblastoma is a GENETIC disease

Genes make up the storybook of life

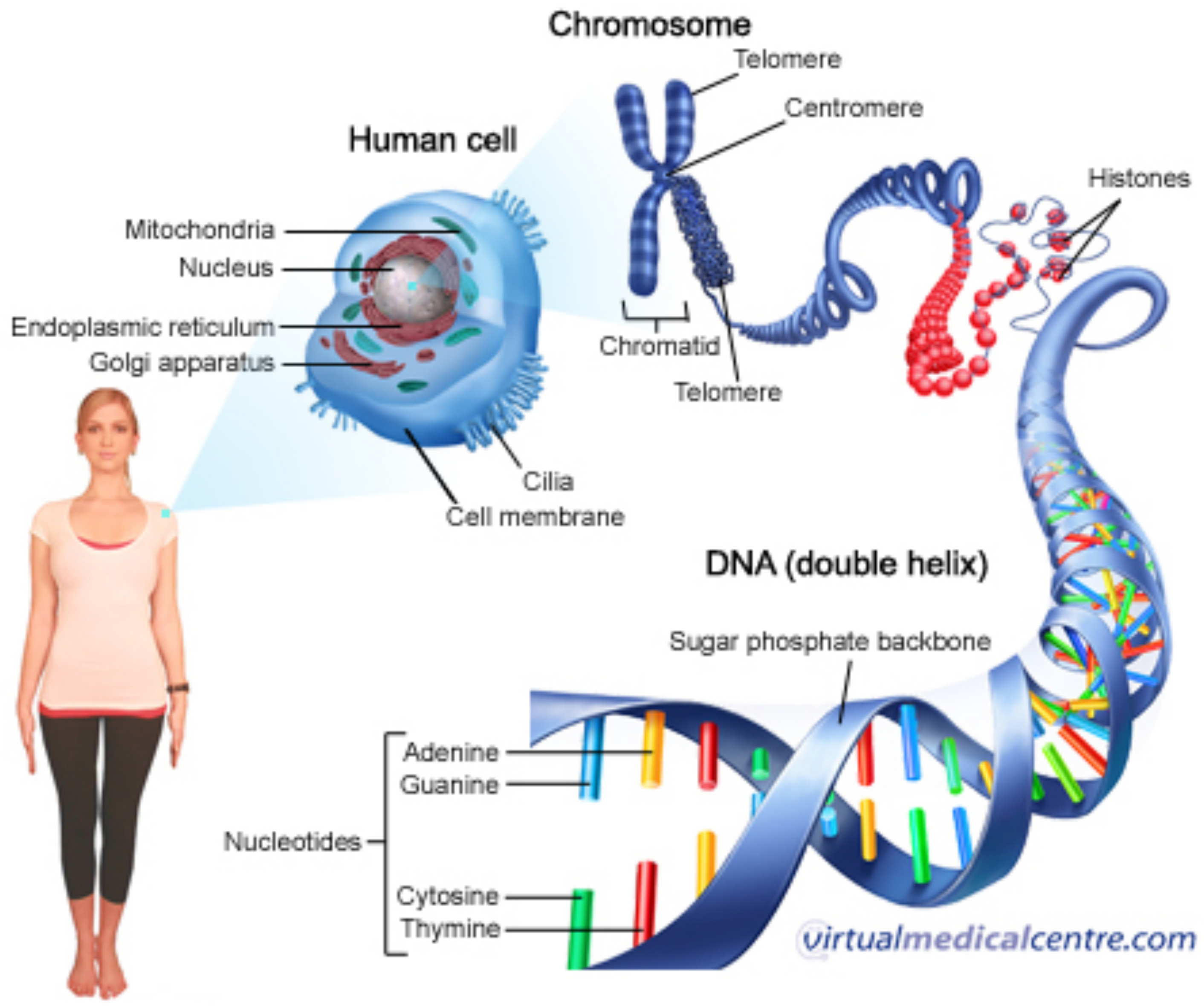

Cancer is a disease of the genome

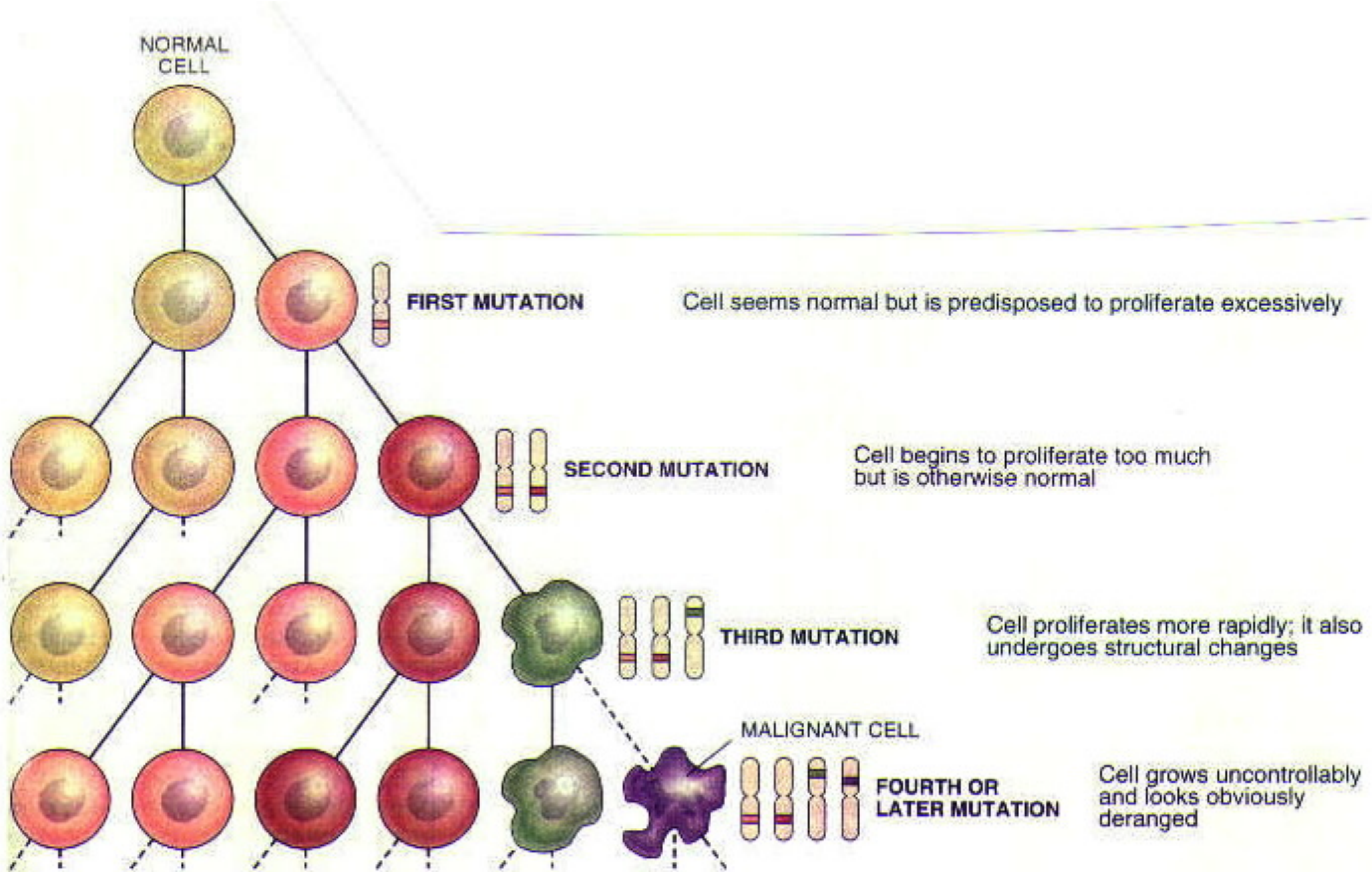

RB1: The Retinoblastoma Tumor Suppressor gene

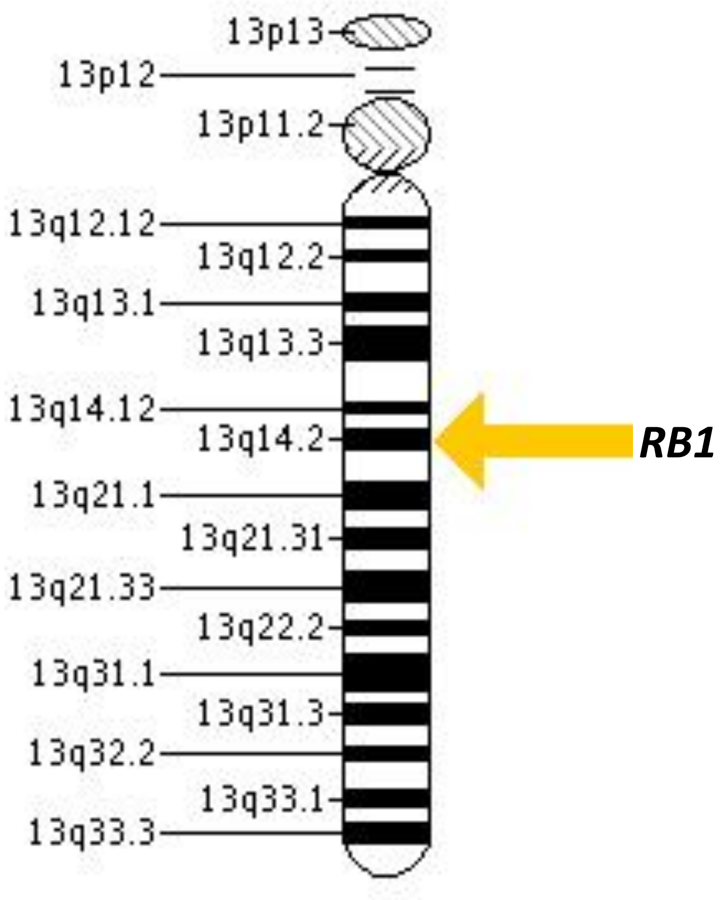

- The *RB1* gene is involved in
  - control of cell growth
  - Keeps DNA healthy

- Mutations in *RB1* initiate retinoblastoma

ALL Retinoblastoma tumours have mutations in both *RB1* copies

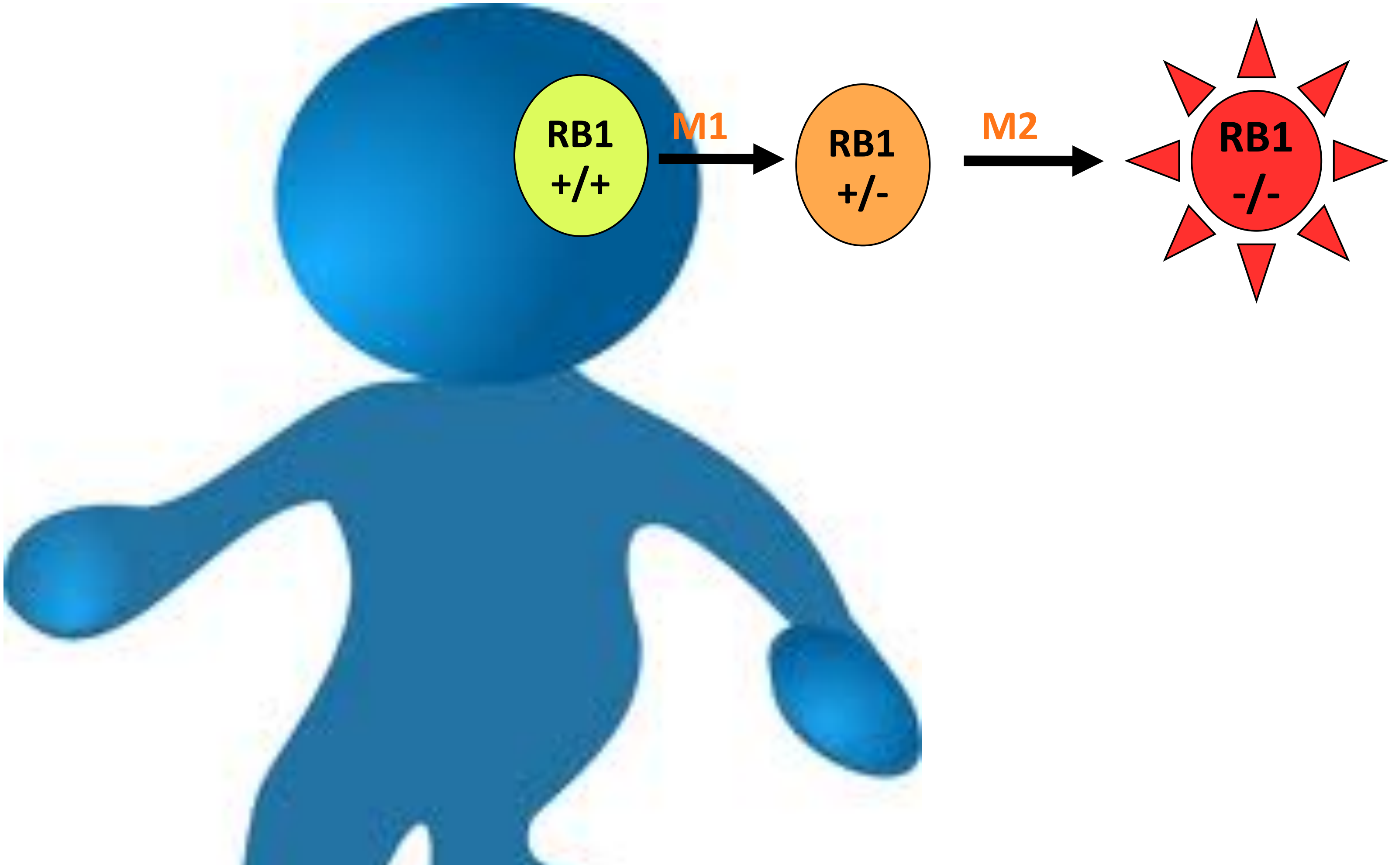

Retinoblastoma is a Genetic Disease

Heritable

All Bilateral

Some unilateral

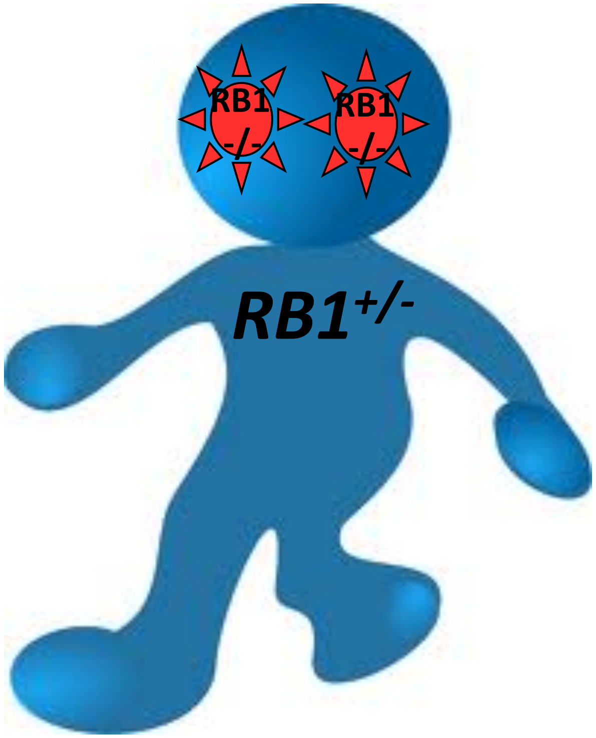

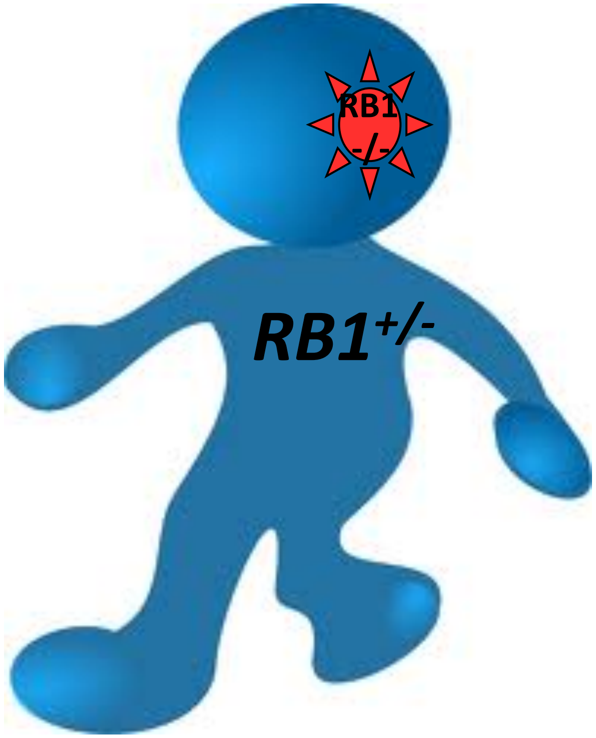

Increased Risk for second cancer

Non-heritable

Most Unilateral

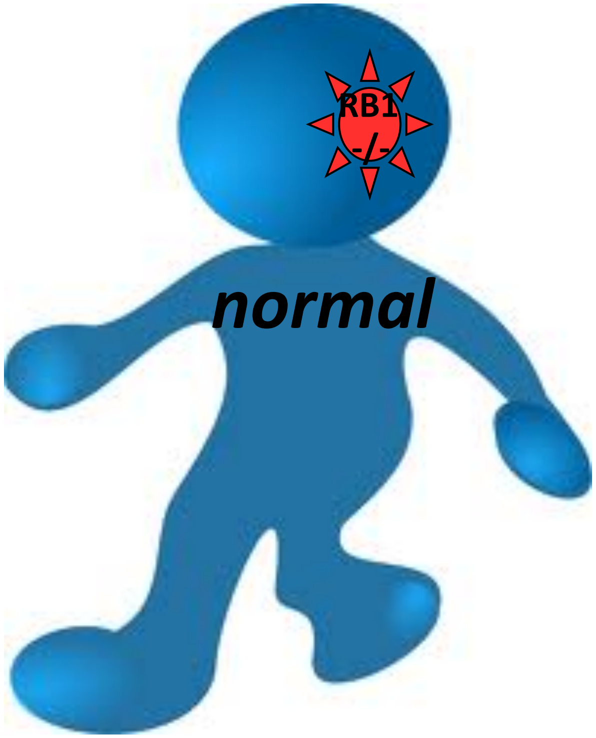

Retinoblastoma can be caused by MYCN-amplification

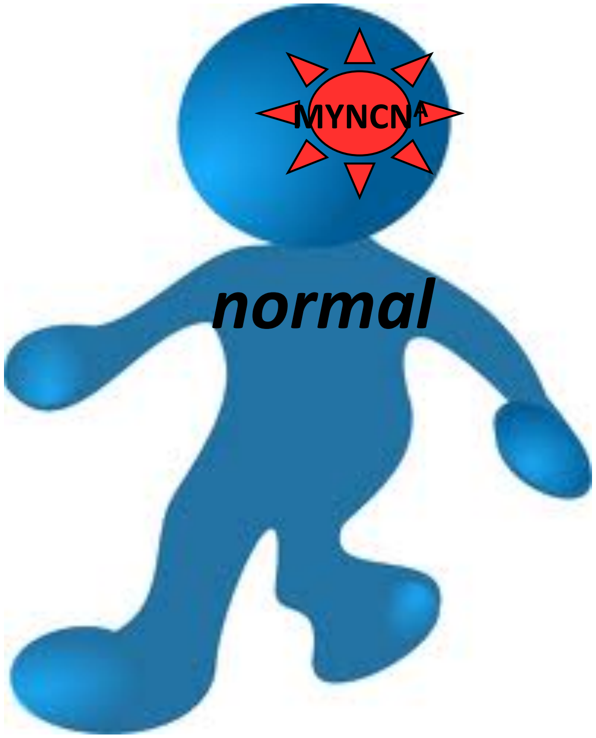

Non-heritable
Always Unilateral
Young age
Neuroblastoma-like pathology

2. Retinoblastoma Genetic Testing

RB1 mutation detection is **critical** part of care

- Risk of multifocal, bilateral disease
- Risk to family members & future offspring
- Risk of second cancers
- Result useful for metastatic surveillance

-Dimaras et al., *Transl Res*, 2010

Distribution of RB1 Germline Mutations

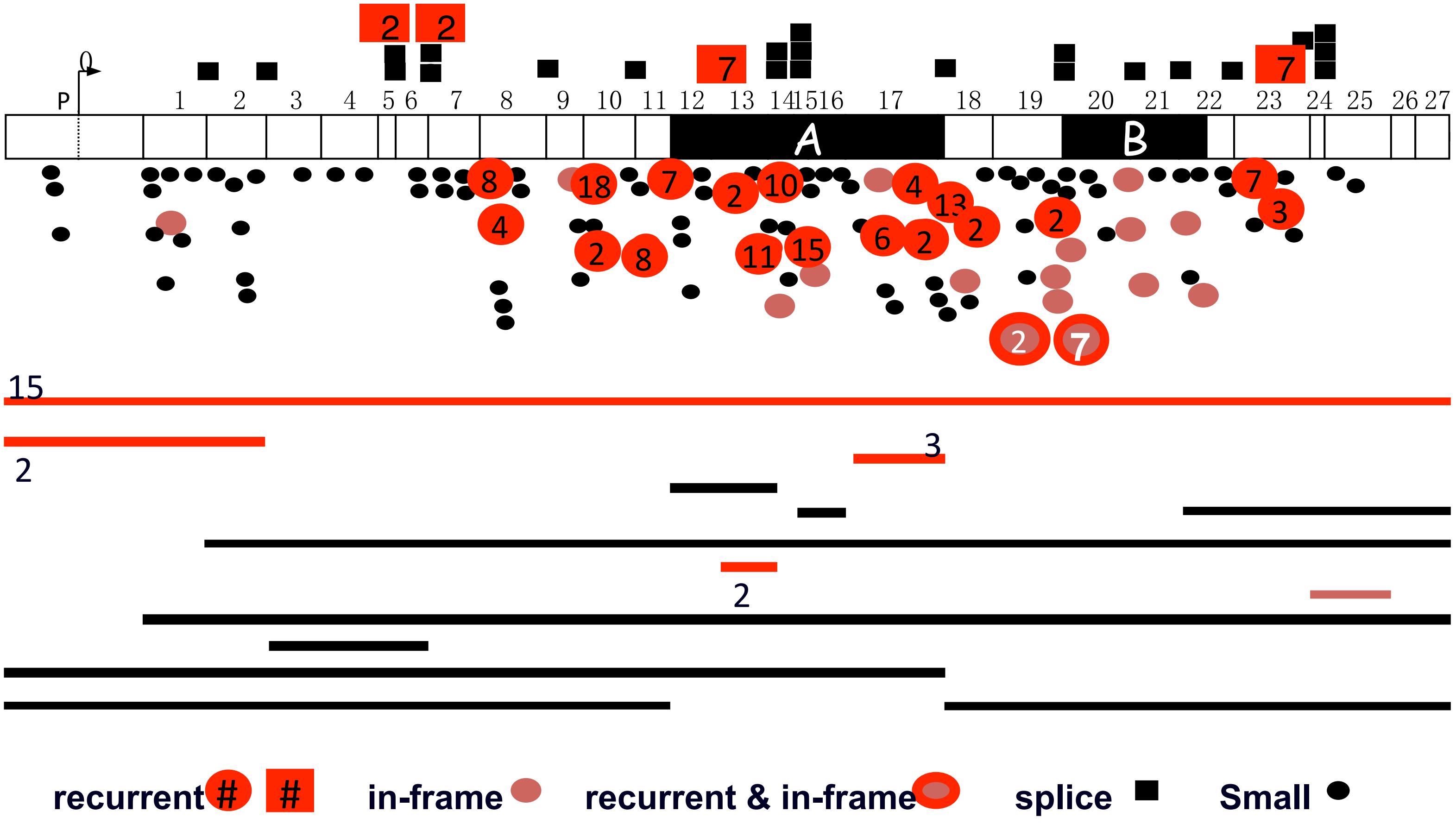

Take Home Message

- With each new patient, we embark on an exploration to find the causative mutation

- There is no short cut.

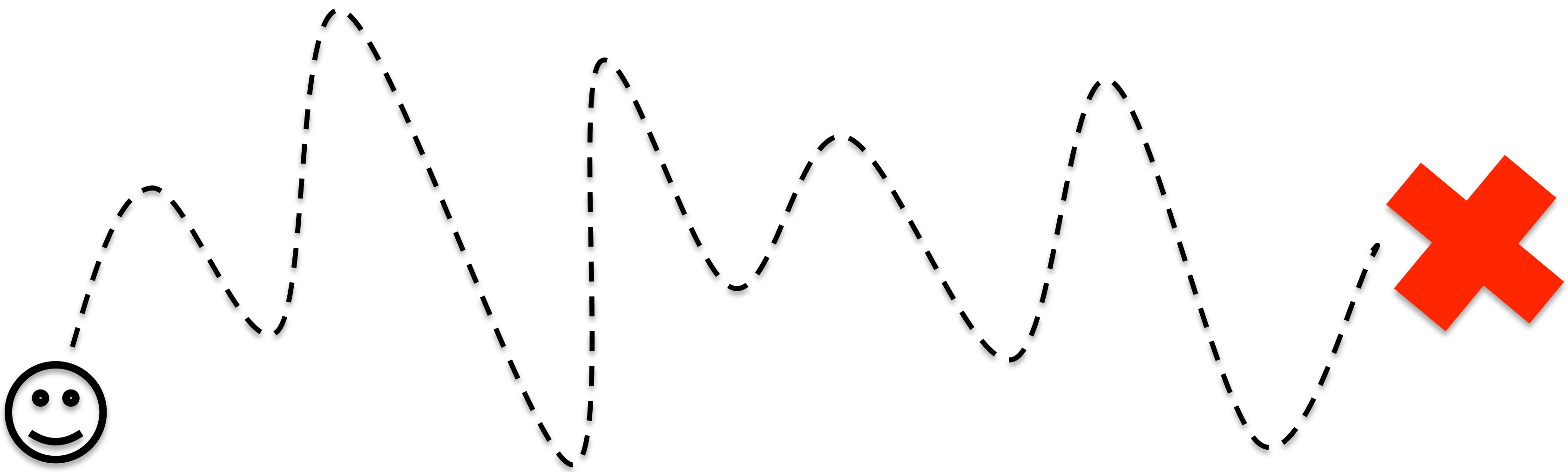

Mutation detection in Proband

- Bilaterals: test blood

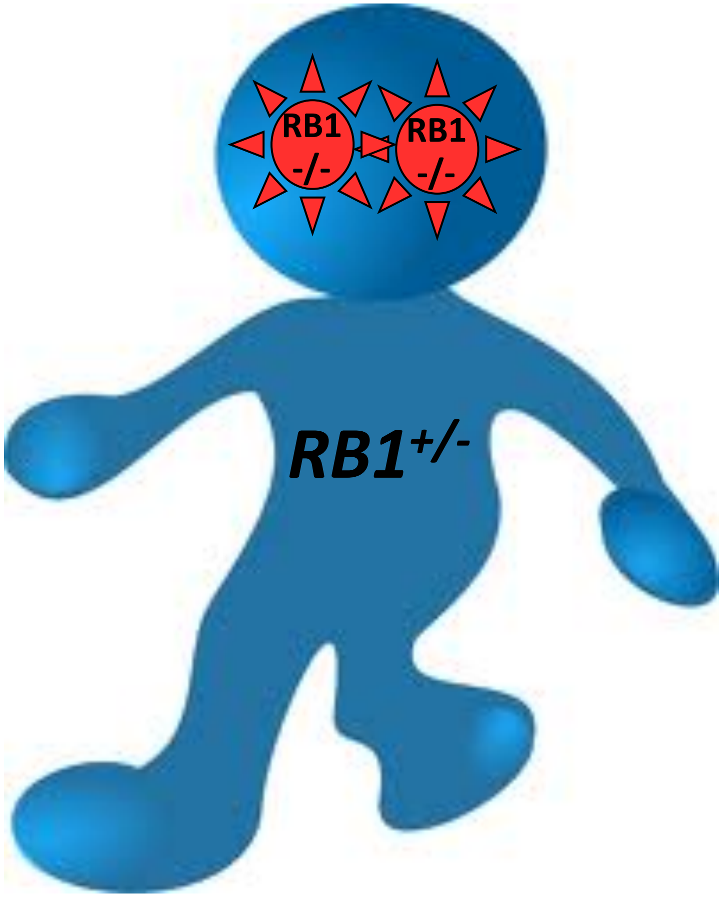

Mutation detection in Proband

- Unilaterals:
  - test tumor to discover mutation(s)
  - then screen blood to see if the mutation is present in germline
  - If no tumor available, test blood

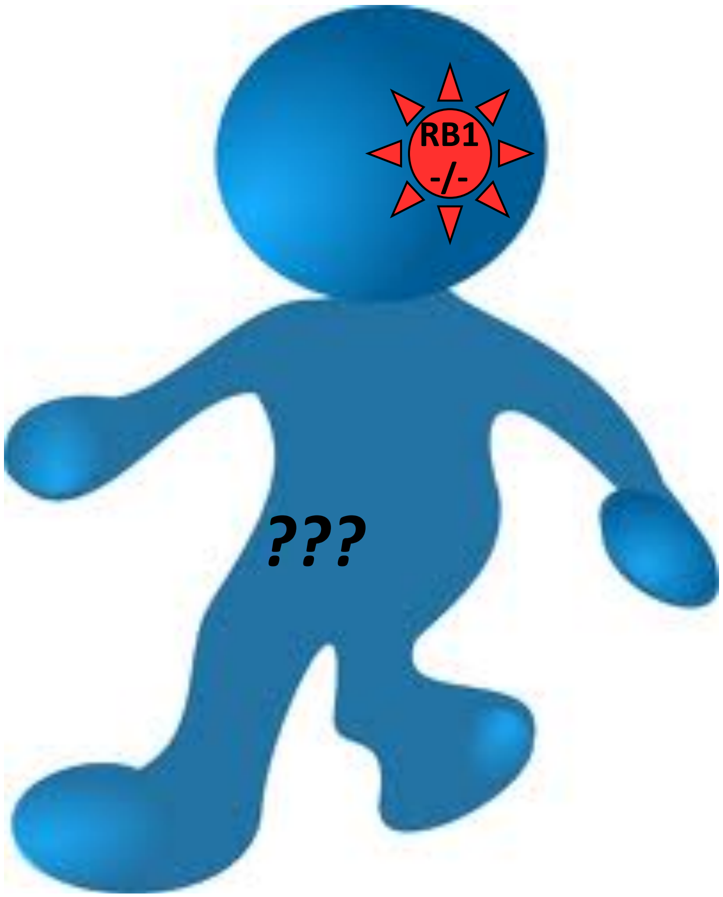

Mosaicism

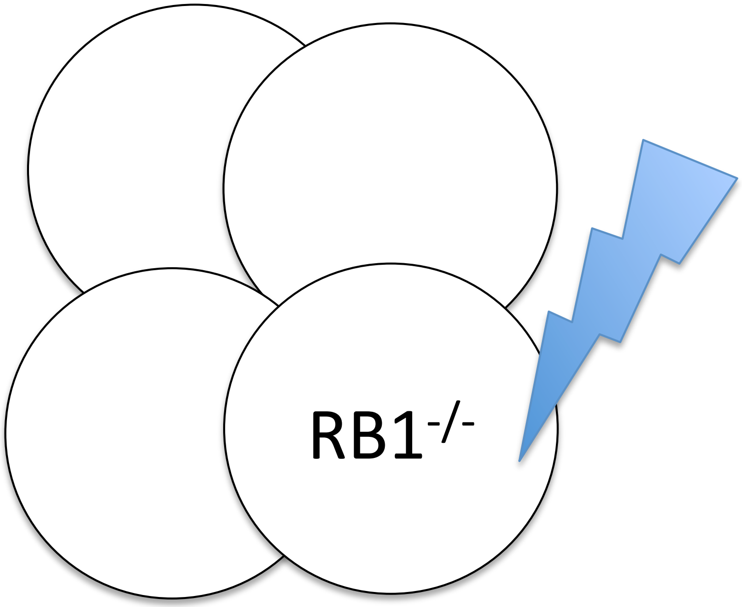

- A proportion of cells in the body carry the *RB1* mutation
- May or may not develop disease
- May or may not be heritable

Low Penetrance RB

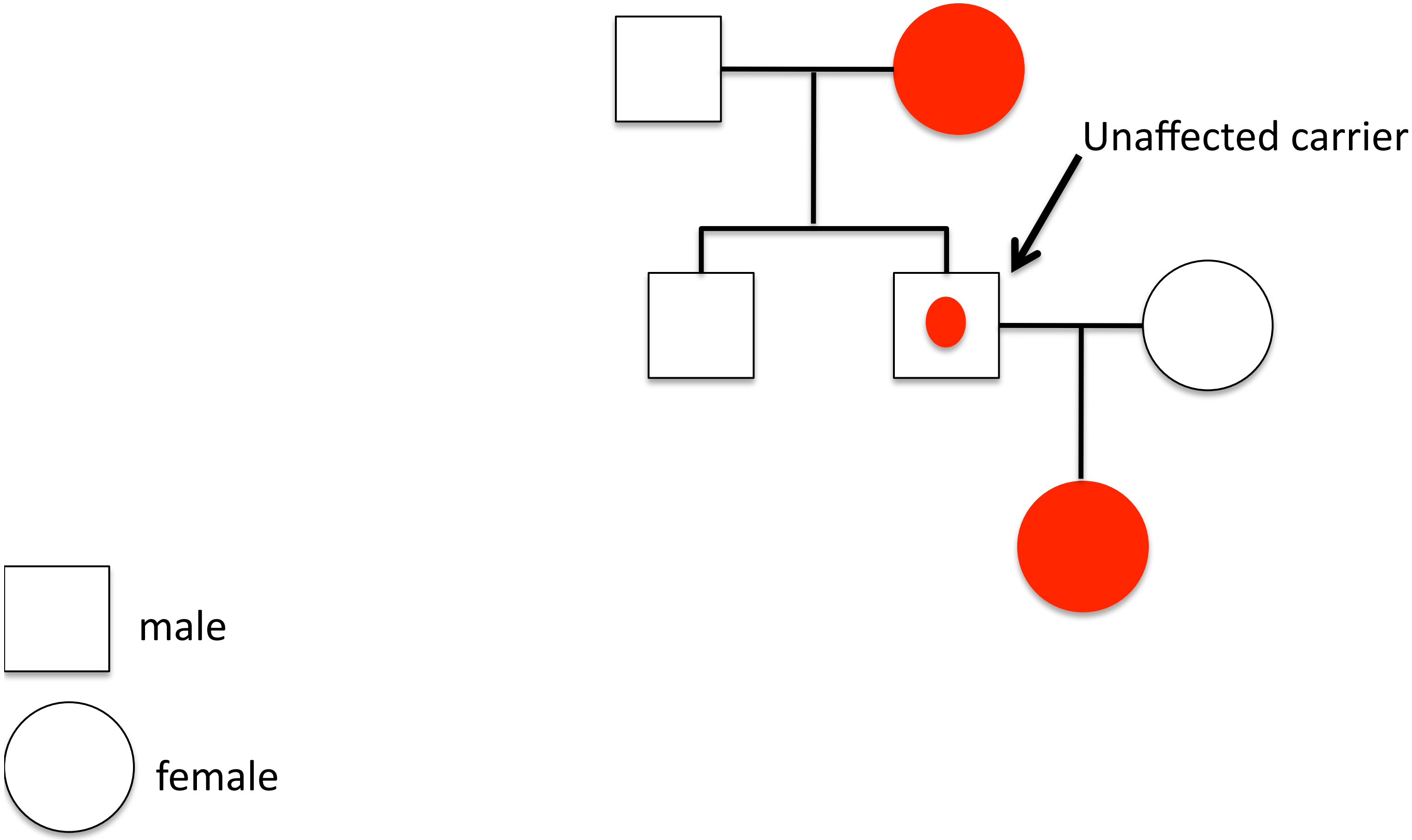

Mutation detection in family

11 bp deletion exon 14

1999

Prenatal Diagnosis

Early Delivery

Laser/Cryo

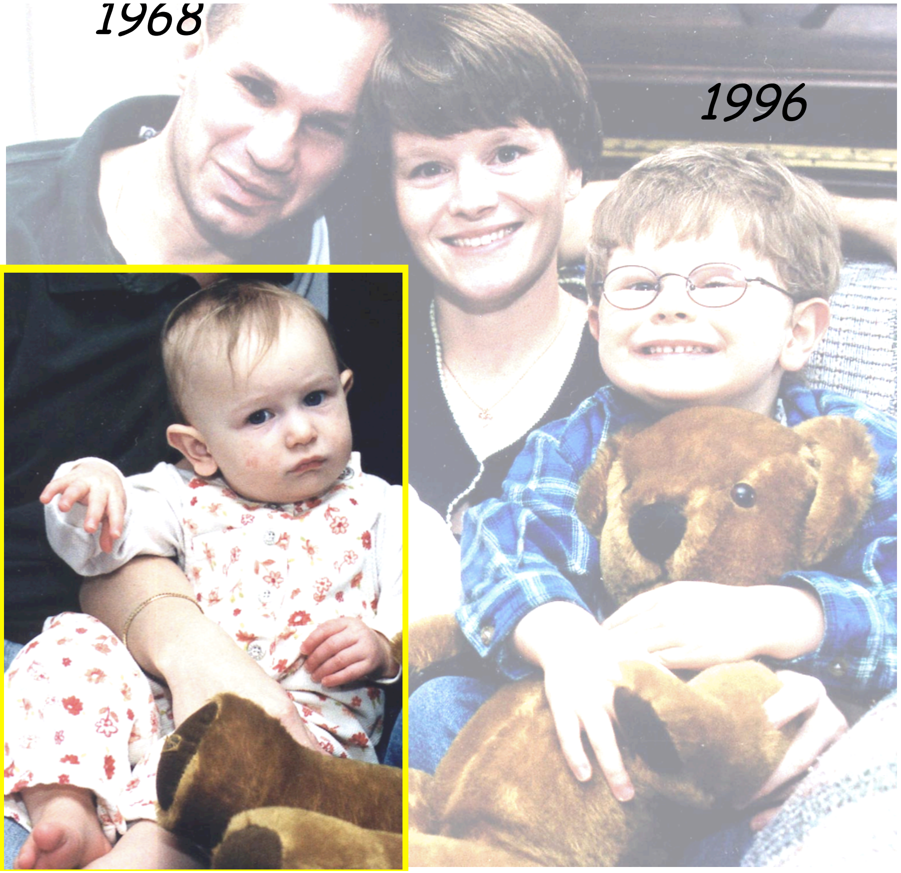

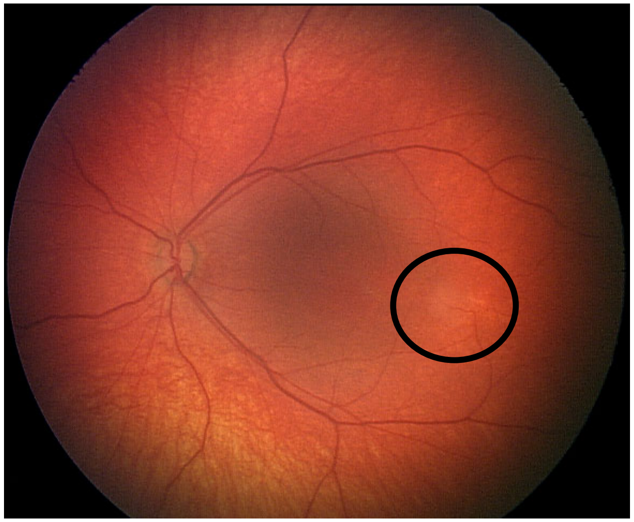

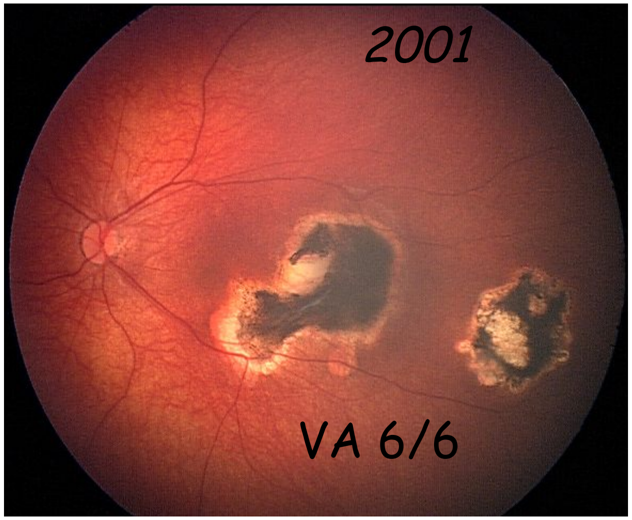

Metastatic Surveillance

- Perform genetic surveillance for evidence of *RB1* mutation in CSF

Non-heritable
Unilateral

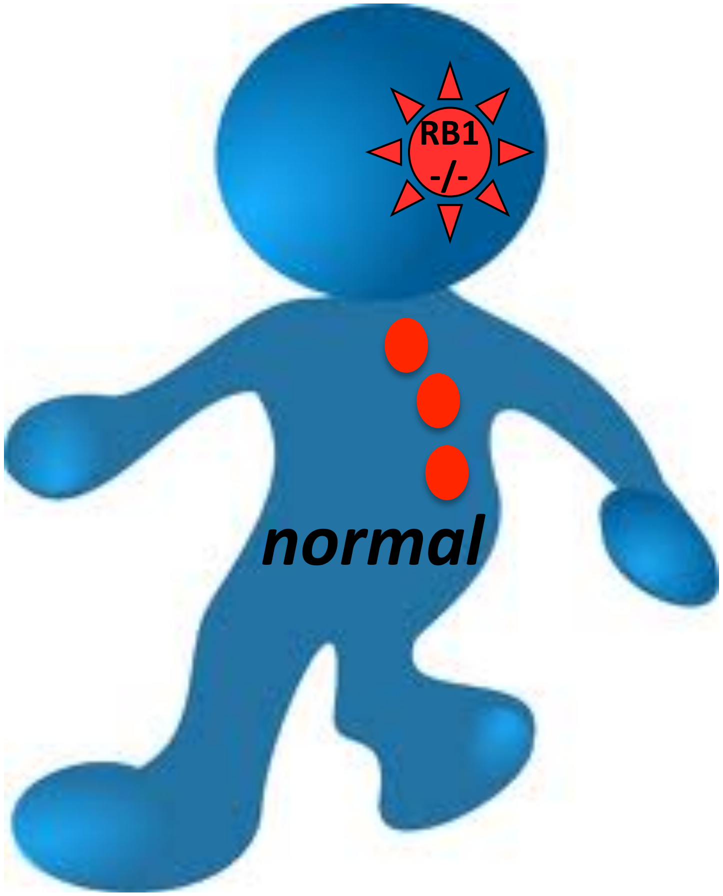

3. Genetic Counseling for Retinoblastoma

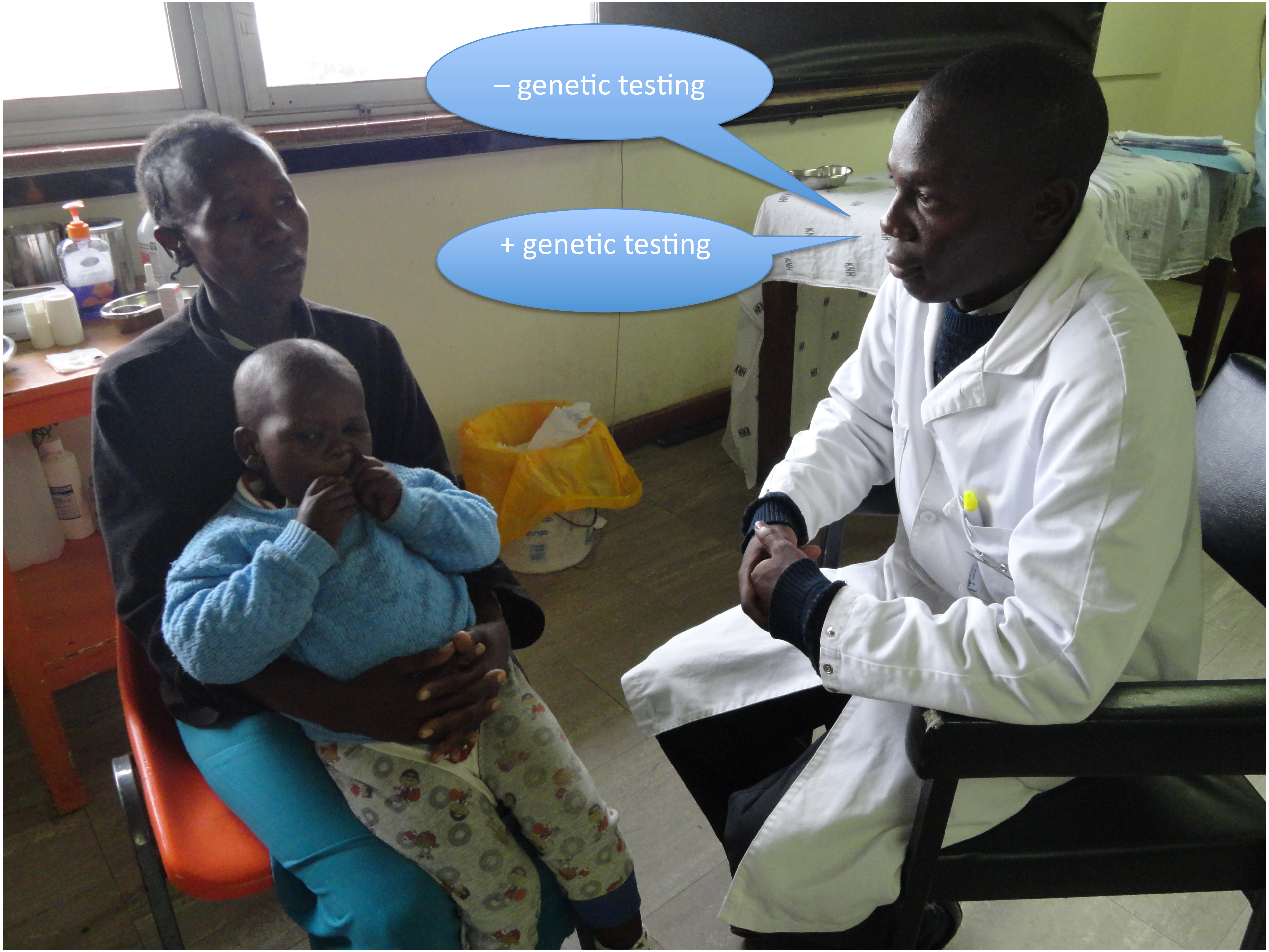
– genetic testing
+ genetic testing

How should genetic counseling for RB take place in Kenya?

- Cultural Context
  - Feelings of guilt are common worldwide in response to genetic testing.
  - Do we understand the specific cultural response to issues of heredity?
  - How can we achieve psychosocial and family support together with genetic counseling?

How should genetic counseling for RB take place in Kenya?

- Differences in Psychosocial needs
  - Do retinoblastoma survivors deal with risk to their child differently from unaffected parents?
- Lay fact and fiction in genetics
  - How do lay people understand genetics?
  - What creative methods can be use to communicate about 'genetics'?

Issues to explore in Kenya

- Physician understanding of genetics
  - Genetics only covered in medical school – no genetic counseling programs
  - What do retinoblastoma healthcare workers understand about genetics?
  - How can they be assisted to meet the needs of patients?
  - Must we train a new class of healthcare workers, to deliver genetic counseling to retinoblastoma families?

  - Is it ethical to provide complex genetic results in absence of local genetic counseling?

Personal Stories: RB Genetics

- How did genetic testing/counseling help you make the best decisions for you and your family?

- How can genetic information best be delivered to survivors and families?

Genetics Breakout Groups

Instructions

- 1. READ. As a group, read through the assigned case study, which includes details of a patient and family affected by retinoblastoma.
- 2. DISCUSS. As a group, discuss the following:
- How would you counsel these family members?
- What are the key concepts regarding genetics and inheritance present in this case?
- If genetic testing were available, what additional information could you learn? Would this change the message to the patient and/or patient family? Would this affect patient care?
- What are some key issues you think should be taken into consideration when counseling patient families and retinoblastoma survivors in Kenya?
- 3. ROLE-PLAY. Act out the counseling session, assigning roles (e.g. parent, survivor, grandparents, doctor, nurse, etc.) to each group member.
- 4. PRESENT. You will have 5 minutes to showcase your presentation to the wider audience.

Case 1

- A 14 month old child presents with leukocoria in the left eye. At the EUA the child is diagnosed with an International Intraocular Retinoblastoma Classification Group E right eye, and normal left eye. The maternal grandmother is also present at diagnosis, and you notice she wears a prosthetic eye. You ask her how she lost her eye, and she says she doesn’t know. You inspect the eyes of both parents and an older sibling of the affected toddler, but their eyes appear normal.

Case 1 Solution

Possibility 1

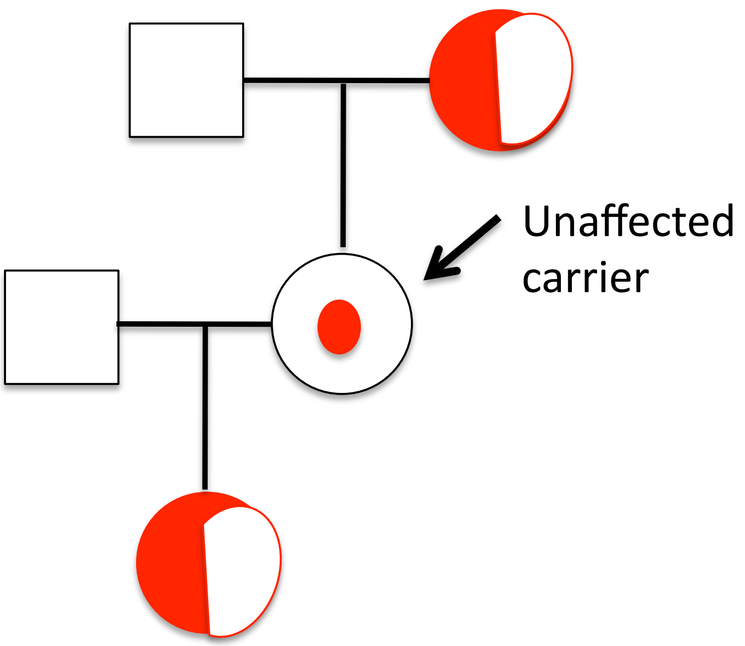

Possibility 2

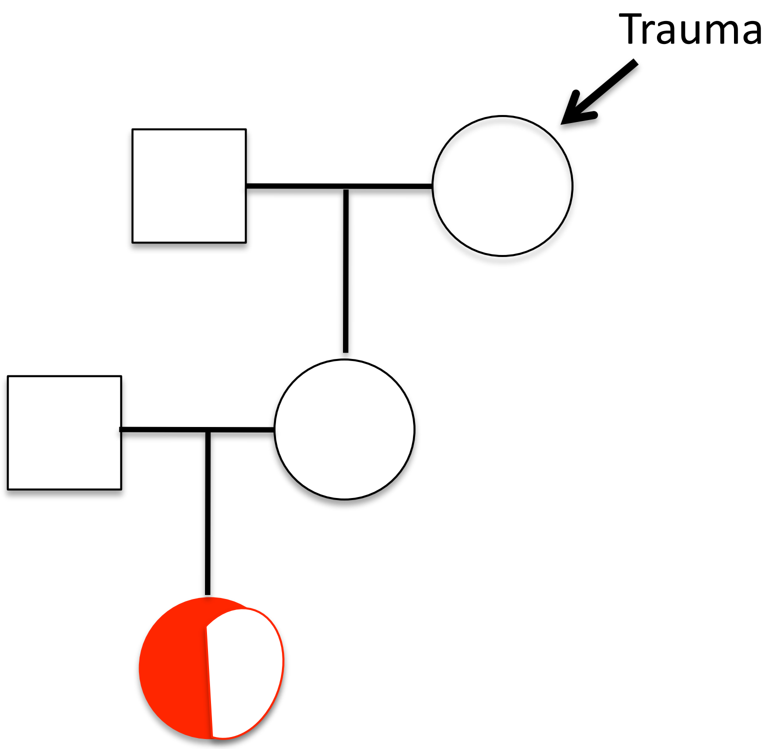

Case 2

- You have been treating Otieno for his bilateral retinoblastoma for a year now. You determined that his right eye was International Intraocular Retinoblastoma Classification Group E and it was enucleated at diagnosis. The left eye was International Intraocular Retinoblastoma Classification Group B and treated by chemotherapy and focal therapy. Otieno’s mother is now pregnant with her second child. She asks you if this baby will get retinoblastoma, and if there is anything you can do to prevent it from happening.

Case 2 Solution

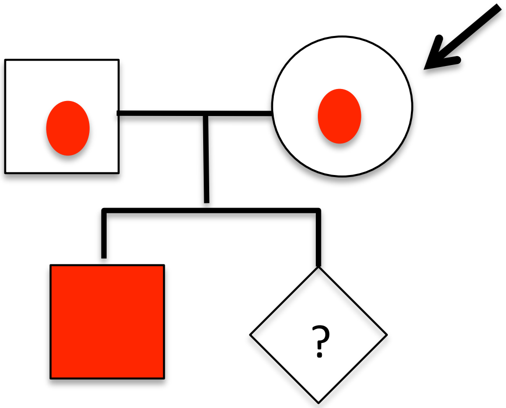

Unaffected carrier? Which one? Maybe none.

Case 3

- A male, 22 years old, had unilateral retinoblastoma as a child. The eye was removed when he was 4 months old and he is a healthy adult today. He is now engaged to be married and is concerned about the retinoblastoma risk to his children.

Case 3 Solution

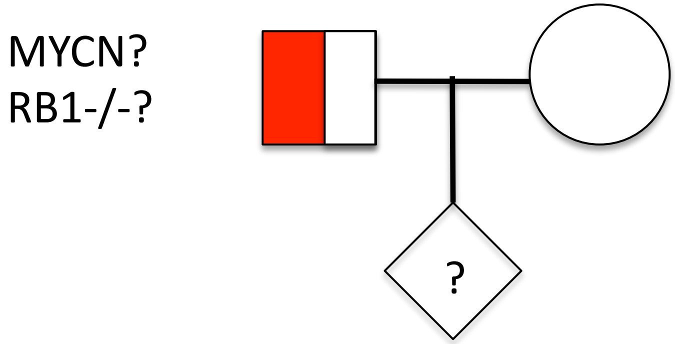

Case 4

- One of your patients is a 20 month old child with unilateral extra-ocular retinoblastoma. The patient has a newborn sibling. The family tells you there is no family history of retinoblastoma.

Case 4 Solution

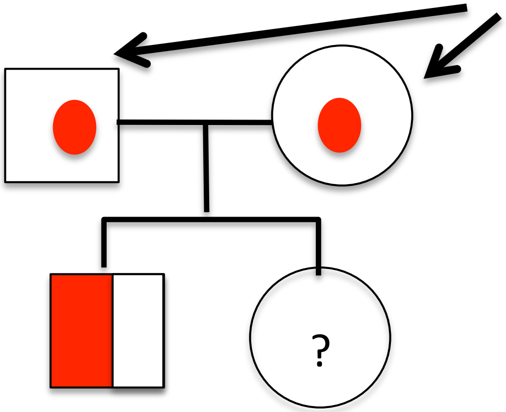

Unaffected carrier parent? Which one?

Case 5

- One of your patients is a 20 month old child with unilateral extraocular retinoblastoma. The patient has a newborn sibling. The family tells you they had two older children who died from retinoblastoma.

Case 5 Solution

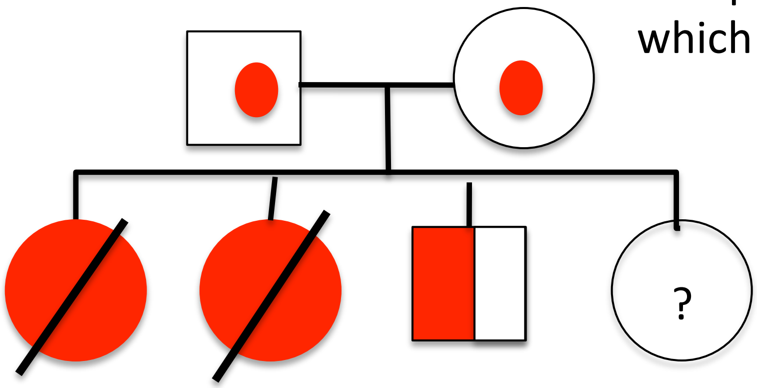

One parent IS unaffected carrier – which one?

Baby definitely at risk (50%)
Genetic testing would confirm family mutation
