# Supplementary material for: Cancer Genetics Education in a Low- to Middle-Income Country: Evaluation of an Interactive Workshop for Clinicians in Kenya
Source: PLoS One. 2015 Jun 2;10(6):e0129852. doi: 10.1371/journal.pone.0129852 (PMC4452713; doi:10.1371/journal.pone.0129852)
Supplement: S3 File — (PDF) [file pone.0129852.s003.pdf]

Case 1.

A 14 month old child presents with leukocoria in the left eye. At the EUA the child is diagnosed with an International Intraocular Retinoblastoma Classification Group E right eye, and normal left eye. The maternal grandmother is also present at diagnosis, and you notice she wears a prosthetic eye. You ask her how she lost her eye, and she says she doesn’t know. You inspect the eyes of both parents and an older sibling of the affected toddler, but their eyes appear normal.

Notes

Case 2.

You have been treating Otieno for his bilateral retinoblastoma for a year now. You determined that his right eye was International Intraocular Retinoblastoma Classification Group E and it was enucleated at diagnosis. The left eye was International Intraocular Retinoblastoma Classification Group B and treated by chemotherapy and focal therapy. Otieno's mother is now pregnant with her second child. She asks you if this baby will get retinoblastoma, and if there is anything you can do to prevent it from happening.

Notes

Case 3.

A male, 22 years old, had unilateral retinoblastoma as a child. The eye was removed when he was 4 months old and he is a healthy adult today. He is now engaged to be married and is concerned about the retinoblastoma risk to his children.

Notes

Case 4

One of your patients is a 20 month old child with unilateral extra-ocular retinoblastoma. The patient has a newborn sibling. The family tells you there is no family history of retinoblastoma.

Notes

Case 5

One of your patients is a 20 month old child with unilateral extraocular retinoblastoma. The patient has a newborn sibling. The family tells you they had two older children who died from retinoblastoma.

Notes
